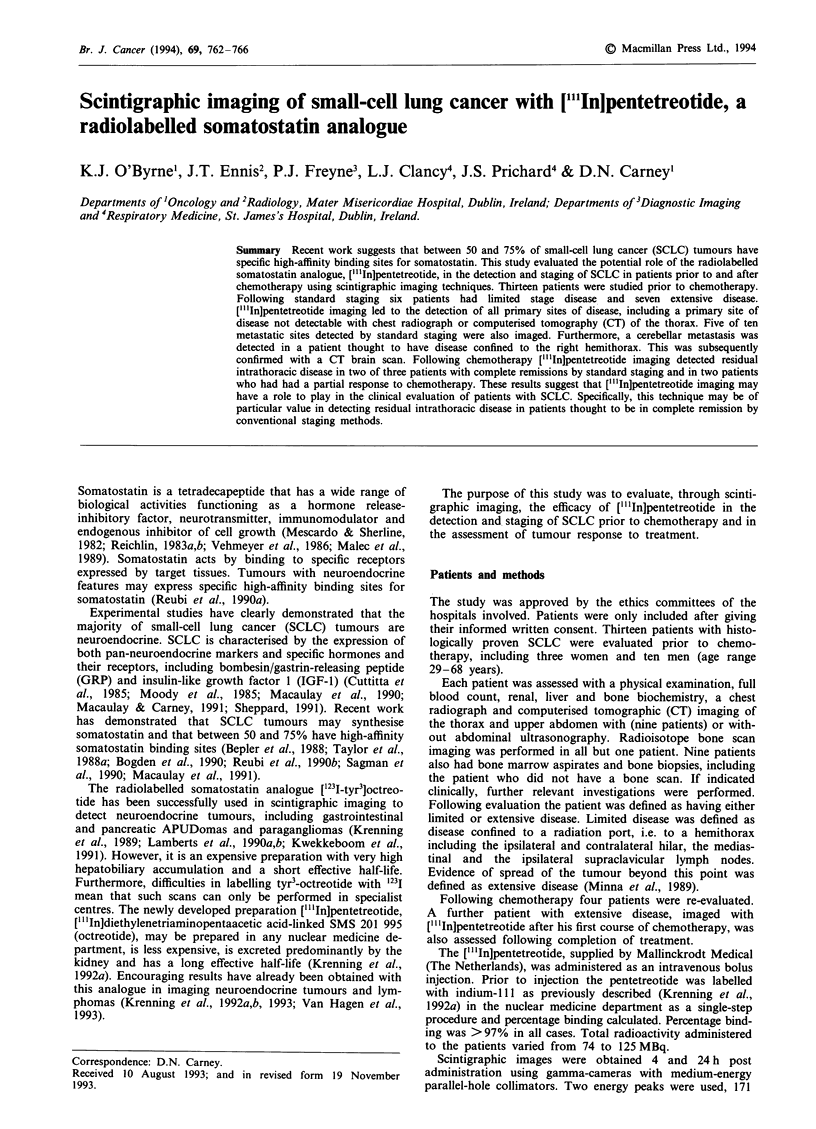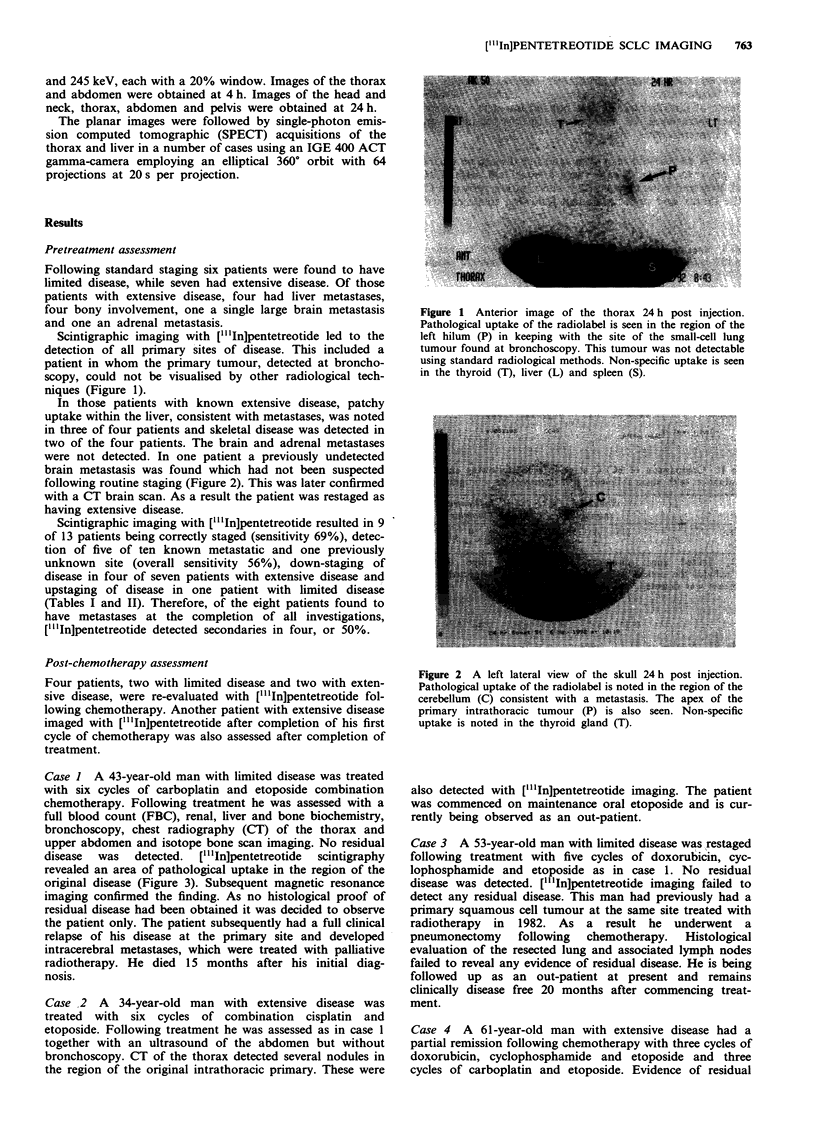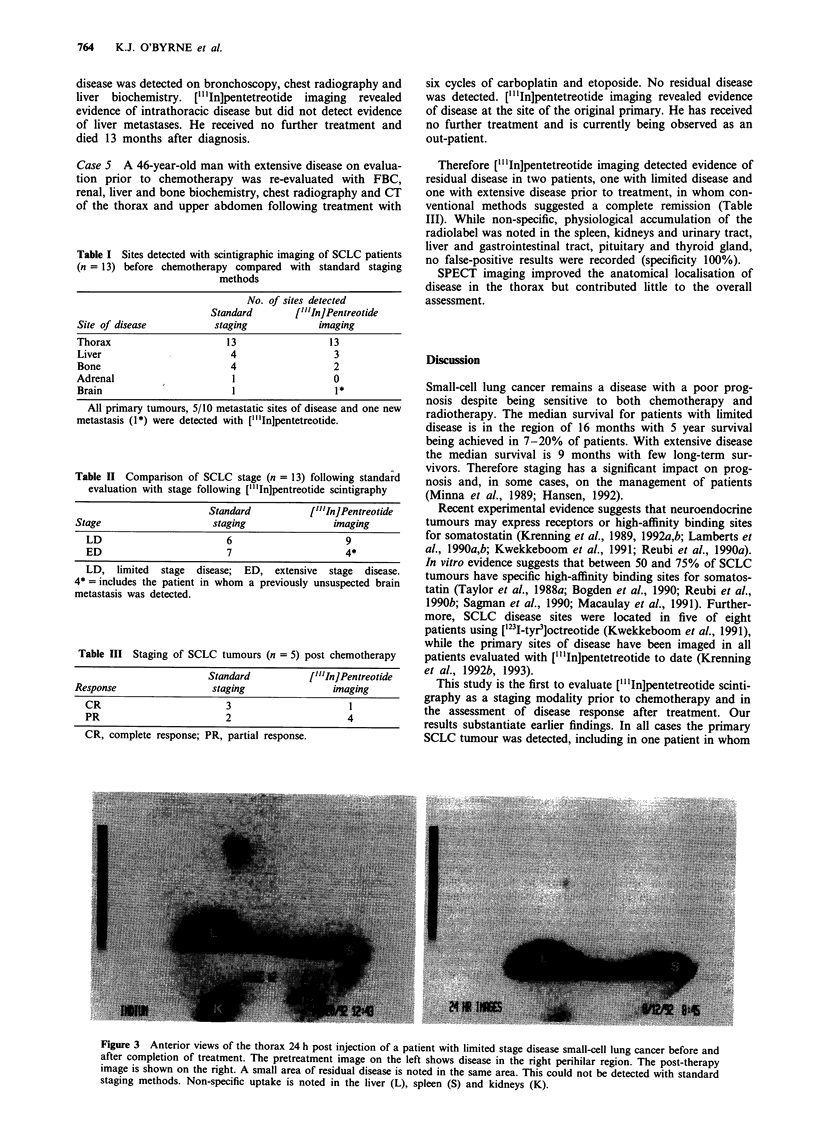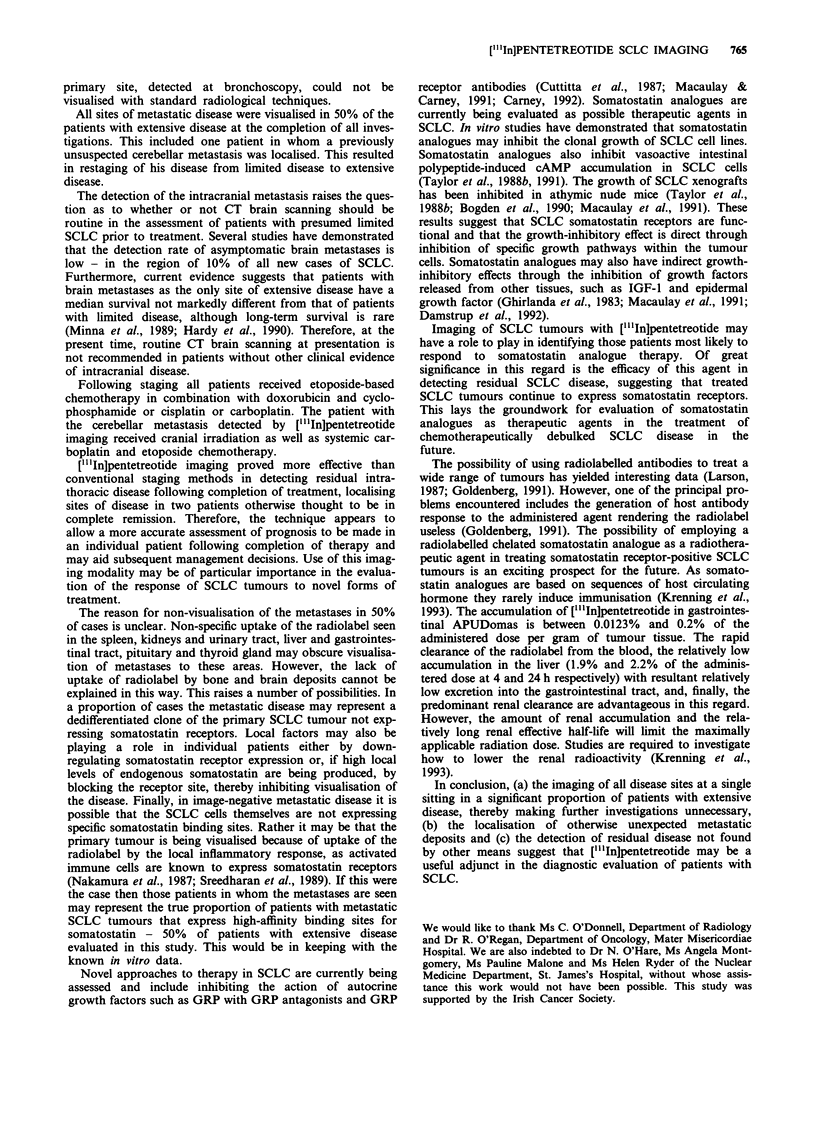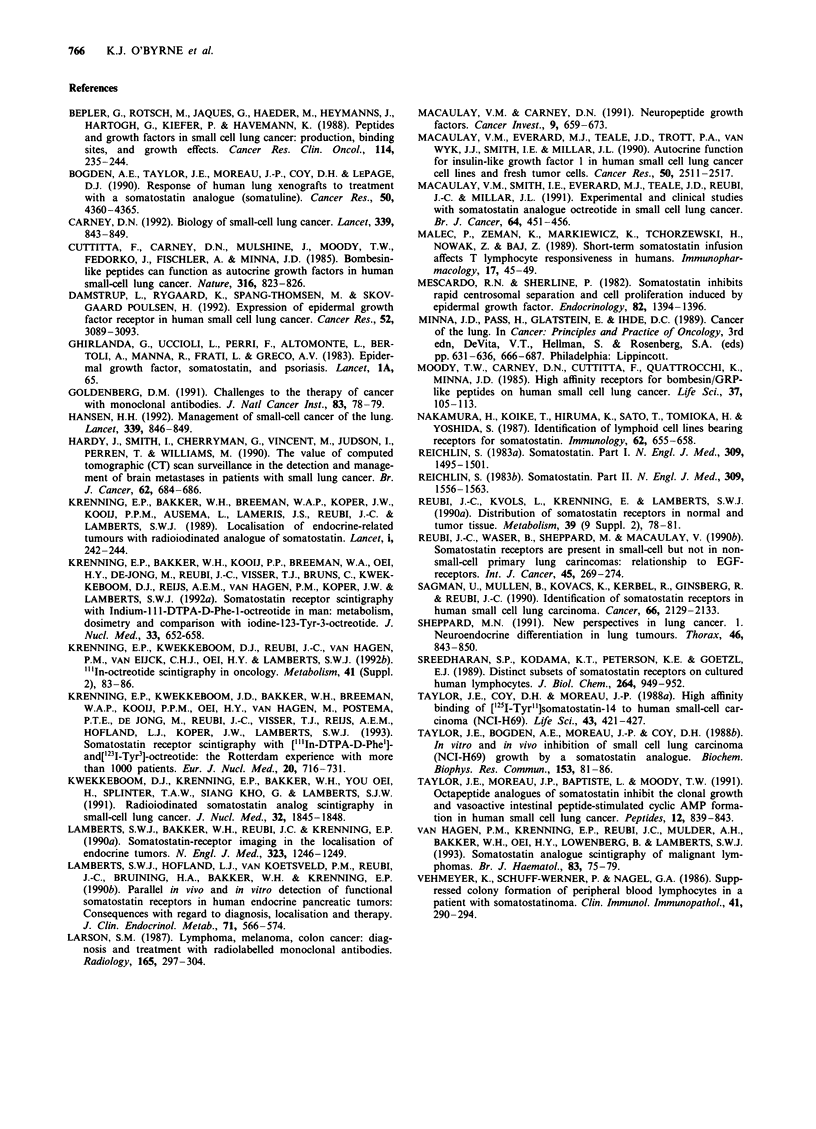# Scintigraphic imaging of small-cell lung cancer with [111In]pentetreotide, a radiolabelled somatostatin analogue.

**DOI:** 10.1038/bjc.1994.144

**Published:** 1994-04

**Authors:** K. J. O'Byrne, J. T. Ennis, P. J. Freyne, L. J. Clancy, J. S. Prichard, D. N. Carney

**Affiliations:** Department of Oncology, Mater Misericordiae Hospital, Dublin, Ireland.

## Abstract

**Images:**


Br. J. Cancer (1994), 69, 762–766 © Macmillan Press Ltd., 1994

### Scintigraphic imaging of small-cell lung cancer with [$^{111}$In]pentetreotide, a radiolabelled somatostatin analogue

K.J. O'Byrne[1], J.T. Ennis[2], P.J. Freyne[3], L.J. Clancy[4], J.S. Prichard[4] & D.N. Carney[1]

*Departments of [1]Oncology and [2]Radiology, Mater Misericordiae Hospital, Dublin, Ireland; Departments of [3]Diagnostic Imaging and [4]Respiratory Medicine, St. James's Hospital, Dublin, Ireland.*

**Summary** Recent work suggests that between 50 and 75% of small-cell lung cancer (SCLC) tumours have specific high-affinity binding sites for somatostatin. This study evaluated the potential role of the radiolabelled somatostatin analogue, [$^{111}$In]pentetreotide, in the detection and staging of SCLC in patients prior to and after chemotherapy using scintigraphic imaging techniques. Thirteen patients were studied prior to chemotherapy. Following standard staging six patients had limited stage disease and seven extensive disease. [$^{111}$In]pentetreotide imaging led to the detection of all primary sites of disease, including a primary site of disease not detectable with chest radiograph or computerised tomography (CT) of the thorax. Five of ten metastatic sites detected by standard staging were also imaged. Furthermore, a cerebellar metastasis was detected in a patient thought to have disease confined to the right hemithorax. This was subsequently confirmed with a CT brain scan. Following chemotherapy [$^{111}$In]pentetreotide imaging detected residual intrathoracic disease in two of three patients with complete remissions by standard staging and in two patients who had had a partial response to chemotherapy. These results suggest that [$^{111}$In]pentetreotide imaging may have a role to play in the clinical evaluation of patients with SCLC. Specifically, this technique may be of particular value in detecting residual intrathoracic disease in patients thought to be in complete remission by conventional staging methods.

Somatostatin is a tetradecapeptide that has a wide range of biological activities functioning as a hormone release-inhibitory factor, neurotransmitter, immunomodulator and endogenous inhibitor of cell growth (Mescardo & Sherline, 1982; Reichlin, 1983*a*,*b*; Vehmeyer *et al*., 1986; Malec *et al*., 1989). Somatostatin acts by binding to specific receptors expressed by target tissues. Tumours with neuroendocrine features may express specific high-affinity binding sites for somatostatin (Reubi *et al*., 1990*a*).

Experimental studies have clearly demonstrated that the majority of small-cell lung cancer (SCLC) tumours are neuroendocrine. SCLC is characterised by the expression of both pan-neuroendocrine markers and specific hormones and their receptors, including bombesin/gastrin-releasing peptide (GRP) and insulin-like growth factor 1 (IGF-1) (Cuttitta *et al*., 1985; Moody *et al*., 1985; Macaulay *et al*., 1990; Macaulay & Carney, 1991; Sheppard, 1991). Recent work has demonstrated that SCLC tumours may synthesise somatostatin and that between 50 and 75% have high-affinity somatostatin binding sites (Bepler *et al*., 1988; Taylor *et al*., 1988*a*; Bogden *et al*., 1990; Reubi *et al*., 1990*b*; Sagman *et al*., 1990; Macaulay *et al*., 1991).

The radiolabelled somatostatin analogue [$^{123}$I-tyr$^{3}$]octreotide has been successfully used in scintigraphic imaging to detect neuroendocrine tumours, including gastrointestinal and pancreatic APUDomas and paragangliomas (Krenning *et al*., 1989; Lamberts *et al*., 1990*a*,*b*; Kwekkeboom *et al*., 1991). However, it is an expensive preparation with very high hepatobiliary accumulation and a short effective half-life. Furthermore, difficulties in labelling tyr$^{3}$-octreotide with $^{123}$I mean that such scans can only be performed in specialist centres. The newly developed preparation [$^{111}$In]pentetreotide, [$^{111}$In]diethylenetriaminopentaacetic acid-linked SMS 201 995 (octreotide), may be prepared in any nuclear medicine department, is less expensive, is excreted predominantly by the kidney and has a long effective half-life (Krenning *et al*., 1992*a*). Encouraging results have already been obtained with this analogue in imaging neuroendocrine tumours and lymphomas (Krenning *et al*., 1992*a*,*b*, 1993; Van Hagen *et al*., 1993).

Correspondence: D.N. Carney.
Received 10 August 1993; and in revised form 19 November 1993.

The purpose of this study was to evaluate, through scintigraphic imaging, the efficacy of [$^{111}$In]pentetreotide in the detection and staging of SCLC prior to chemotherapy and in the assessment of tumour response to treatment.

### Patients and methods

The study was approved by the ethics committees of the hospitals involved. Patients were only included after giving their informed written consent. Thirteen patients with histologically proven SCLC were evaluated prior to chemotherapy, including three women and ten men (age range 29–68 years).

Each patient was assessed with a physical examination, full blood count, renal, liver and bone biochemistry, a chest radiograph and computerised tomographic (CT) imaging of the thorax and upper abdomen with (nine patients) or without abdominal ultrasonography. Radioisotope bone scan imaging was performed in all but one patient. Nine patients also had bone marrow aspirates and bone biopsies, including the patient who did not have a bone scan. If indicated clinically, further relevant investigations were performed. Following evaluation the patient was defined as having either limited or extensive disease. Limited disease was defined as disease confined to a radiation port, i.e. to a hemithorax including the ipsilateral and contralateral hilar, the mediastinal and the ipsilateral supraclavicular lymph nodes. Evidence of spread of the tumour beyond this point was defined as extensive disease (Minna *et al*., 1989).

Following chemotherapy four patients were re-evaluated. A further patient with extensive disease, imaged with [$^{111}$In]pentetreotide after his first course of chemotherapy, was also assessed following completion of treatment.

The [$^{111}$In]pentetreotide, supplied by Mallinckrodt Medical (The Netherlands), was administered as an intravenous bolus injection. Prior to injection the pentetreotide was labelled with indium-111 as previously described (Krenning *et al*., 1992*a*) in the nuclear medicine department as a single-step procedure and percentage binding calculated. Percentage binding was $>97\%$ in all cases. Total radioactivity administered to the patients varied from 74 to 125 MBq.

Scintigraphic images were obtained 4 and 24 h post administration using gamma-cameras with medium-energy parallel-hole collimators. Two energy peaks were used, 171

and 245 keV, each with a 20% window. Images of the thorax and abdomen were obtained at 4 h. Images of the head and neck, thorax, abdomen and pelvis were obtained at 24 h.

The planar images were followed by single-photon emission computed tomographic (SPECT) acquisitions of the thorax and liver in a number of cases using an IGE 400 ACT gamma-camera employing an elliptical 360° orbit with 64 projections at 20 s per projection.

### Results

*Pretreatment assessment*

Following standard staging six patients were found to have limited disease, while seven had extensive disease. Of those patients with extensive disease, four had liver metastases, four bony involvement, one a single large brain metastasis and one an adrenal metastasis.

Scintigraphic imaging with [111In]pentetreotide led to the detection of all primary sites of disease. This included a patient in whom the primary tumour, detected at bronchoscopy, could not be visualised by other radiological techniques (Figure 1).

In those patients with known extensive disease, patchy uptake within the liver, consistent with metastases, was noted in three of four patients and skeletal disease was detected in two of the four patients. The brain and adrenal metastases were not detected. In one patient a previously undetected brain metastasis was found which had not been suspected following routine staging (Figure 2). This was later confirmed with a CT brain scan. As a result the patient was restaged as having extensive disease.

Scintigraphic imaging with [111In]pentetreotide resulted in 9 of 13 patients being correctly staged (sensitivity 69%), detection of five of ten known metastatic and one previously unknown site (overall sensitivity 56%), down-staging of disease in four of seven patients with extensive disease and upstaging of disease in one patient with limited disease (Tables I and II). Therefore, of the eight patients found to have metastases at the completion of all investigations, [111In]pentetreotide detected secondaries in four, or 50%.

**Figure 1** Anterior image of the thorax 24 h post injection. Pathological uptake of the radiolabel is seen in the region of the left hilum (P) in keeping with the site of the small-cell lung tumour found at bronchoscopy. This tumour was not detectable using standard radiological methods. Non-specific uptake is seen in the thyroid (T), liver (L) and spleen (S).

**Figure 2** A left lateral view of the skull 24 h post injection. Pathological uptake of the radiolabel is noted in the region of the cerebellum (C) consistent with a metastasis. The apex of the primary intrathoracic tumour (P) is also seen. Non-specific uptake is noted in the thyroid gland (T).

*Post-chemotherapy assessment*

Four patients, two with limited disease and two with extensive disease, were re-evaluated with [111In]pentetreotide following chemotherapy. Another patient with extensive disease imaged with [111In]pentetreotide after completion of his first cycle of chemotherapy was also assessed after completion of treatment.

*Case 1* A 43-year-old man with limited disease was treated with six cycles of carboplatin and etoposide combination chemotherapy. Following treatment he was assessed with a full blood count (FBC), renal, liver and bone biochemistry, bronchoscopy, chest radiography (CT) of the thorax and upper abdomen and isotope bone scan imaging. No residual disease was detected. [111In]pentetreotide scintigraphy revealed an area of pathological uptake in the region of the original disease (Figure 3). Subsequent magnetic resonance imaging confirmed the finding. As no histological proof of residual disease had been obtained it was decided to observe the patient only. The patient subsequently had a full clinical relapse of his disease at the primary site and developed intracerebral metastases, which were treated with palliative radiotherapy. He died 15 months after his initial diagnosis.

*Case 2* A 34-year-old man with extensive disease was treated with six cycles of combination cisplatin and etoposide. Following treatment he was assessed as in case 1 together with an ultrasound of the abdomen but without bronchoscopy. CT of the thorax detected several nodules in the region of the original intrathoracic primary. These were also detected with [111In]pentetreotide imaging. The patient was commenced on maintenance oral etoposide and is currently being observed as an out-patient.

*Case 3* A 53-year-old man with limited disease was restaged following treatment with five cycles of doxorubicin, cyclophosphamide and etoposide as in case 1. No residual disease was detected. [111In]pentetreotide imaging failed to detect any residual disease. This man had previously had a primary squamous cell tumour at the same site treated with radiotherapy in 1982. As a result he underwent a pneumonectomy following chemotherapy. Histological evaluation of the resected lung and associated lymph nodes failed to reveal any evidence of residual disease. He is being followed up as an out-patient at present and remains clinically disease free 20 months after commencing treatment.

*Case 4* A 61-year-old man with extensive disease had a partial remission following chemotherapy with three cycles of doxorubicin, cyclophosphamide and etoposide and three cycles of carboplatin and etoposide. Evidence of residual disease was detected on bronchoscopy, chest radiography and liver biochemistry. [$^{111}$In]pentetreotide imaging revealed evidence of intrathoracic disease but did not detect evidence of liver metastases. He received no further treatment and died 13 months after diagnosis.

*Case 5* A 46-year-old man with extensive disease on evaluation prior to chemotherapy was re-evaluated with FBC, renal, liver and bone biochemistry, chest radiography and CT of the thorax and upper abdomen following treatment with six cycles of carboplatin and etoposide. No residual disease was detected. [$^{111}$In]pentetreotide imaging revealed evidence of disease at the site of the original primary. He has received no further treatment and is currently being observed as an out-patient.

Therefore [$^{111}$In]pentetreotide imaging detected evidence of residual disease in two patients, one with limited disease and one with extensive disease prior to treatment, in whom conventional methods suggested a complete remission (Table III). While non-specific, physiological accumulation of the radiolabel was noted in the spleen, kidneys and urinary tract, liver and gastrointestinal tract, pituitary and thyroid gland, no false-positive results were recorded (specificity 100%).

SPECT imaging improved the anatomical localisation of disease in the thorax but contributed little to the overall assessment.

**Table I** Sites detected with scintigraphic imaging of SCLC patients ($n$ = 13) before chemotherapy compared with standard staging methods

| | *No. of sites detected* | |
|---|---|---|
| *Site of disease* | *Standard staging* | *[$^{111}$In]Pentreotide imaging* |
| Thorax | 13 | 13 |
| Liver | 4 | 3 |
| Bone | 4 | 2 |
| Adrenal | 1 | 0 |
| Brain | 1 | 1* |

All primary tumours, 5/10 metastatic sites of disease and one new metastasis (1*) were detected with [$^{111}$In]pentetreotide.

**Table II** Comparison of SCLC stage ($n$ = 13) following standard evaluation with stage following [$^{111}$In]pentreotide scintigraphy

| *Stage* | *Standard staging* | *[$^{111}$In]Pentreotide imaging* |
|---|---|---|
| LD | 6 | 9 |
| ED | 7 | 4* |

LD, limited stage disease; ED, extensive stage disease. 4* = includes the patient in whom a previously unsuspected brain metastasis was detected.

**Table III** Staging of SCLC tumours ($n$ = 5) post chemotherapy

| *Response* | *Standard staging* | *[$^{111}$In]Pentreotide imaging* |
|---|---|---|
| CR | 3 | 1 |
| PR | 2 | 4 |

CR, complete response; PR, partial response.

### Discussion

Small-cell lung cancer remains a disease with a poor prognosis despite being sensitive to both chemotherapy and radiotherapy. The median survival for patients with limited disease is in the region of 16 months with 5 year survival being achieved in 7–20% of patients. With extensive disease the median survival is 9 months with few long-term survivors. Therefore staging has a significant impact on prognosis and, in some cases, on the management of patients (Minna *et al.*, 1989; Hansen, 1992).

Recent experimental evidence suggests that neuroendocrine tumours may express receptors or high-affinity binding sites for somatostatin (Krenning *et al.*, 1989, 1992*a*,*b*; Lamberts *et al.*, 1990*a*,*b*; Kwekkeboom *et al.*, 1991; Reubi *et al.*, 1990*a*). *In vitro* evidence suggests that between 50 and 75% of SCLC tumours have specific high-affinity binding sites for somatostatin (Taylor *et al.*, 1988*a*; Bogden *et al.*, 1990; Reubi *et al.*, 1990*b*; Sagman *et al.*, 1990; Macaulay *et al.*, 1991). Furthermore, SCLC disease sites were located in five of eight patients using [$^{123}$I-tyr$^{3}$]octreotide (Kwekkeboom *et al.*, 1991), while the primary sites of disease have been imaged in all patients evaluated with [$^{111}$In]pentetreotide to date (Krenning *et al.*, 1992*b*, 1993).

This study is the first to evaluate [$^{111}$In]pentetreotide scintigraphy as a staging modality prior to chemotherapy and in the assessment of disease response after treatment. Our results substantiate earlier findings. In all cases the primary SCLC tumour was detected, including in one patient in whom

**Figure 3** Anterior views of the thorax 24 h post injection of a patient with limited stage disease small-cell lung cancer before and after completion of treatment. The pretreatment image on the left shows disease in the right perihilar region. The post-therapy image is shown on the right. A small area of residual disease is noted in the same area. This could not be detected with standard staging methods. Non-specific uptake is noted in the liver (L), spleen (S) and kidneys (K).

primary site, detected at bronchoscopy, could not be visualised with standard radiological techniques.

All sites of metastatic disease were visualised in 50% of the patients with extensive disease at the completion of all investigations. This included one patient in whom a previously unsuspected cerebellar metastasis was localised. This resulted in restaging of his disease from limited disease to extensive disease.

The detection of the intracranial metastasis raises the question as to whether or not CT brain scanning should be routine in the assessment of patients with presumed limited SCLC prior to treatment. Several studies have demonstrated that the detection rate of asymptomatic brain metastases is low – in the region of 10% of all new cases of SCLC. Furthermore, current evidence suggests that patients with brain metastases as the only site of extensive disease have a median survival not markedly different from that of patients with limited disease, although long-term survival is rare (Minna *et al.*, 1989; Hardy *et al.*, 1990). Therefore, at the present time, routine CT brain scanning at presentation is not recommended in patients without other clinical evidence of intracranial disease.

Following staging all patients received etoposide-based chemotherapy in combination with doxorubicin and cyclophosphamide or cisplatin or carboplatin. The patient with the cerebellar metastasis detected by [$^{111}$In]pentetreotide imaging received cranial irradiation as well as systemic carboplatin and etoposide chemotherapy.

[$^{111}$In]pentetreotide imaging proved more effective than conventional staging methods in detecting residual intrathoracic disease following completion of treatment, localising sites of disease in two patients otherwise thought to be in complete remission. Therefore, the technique appears to allow a more accurate assessment of prognosis to be made in an individual patient following completion of therapy and may aid subsequent management decisions. Use of this imaging modality may be of particular importance in the evaluation of the response of SCLC tumours to novel forms of treatment.

The reason for non-visualisation of the metastases in 50% of cases is unclear. Non-specific uptake of the radiolabel seen in the spleen, kidneys and urinary tract, liver and gastrointestinal tract, pituitary and thyroid gland may obscure visualisation of metastases to these areas. However, the lack of uptake of radiolabel by bone and brain deposits cannot be explained in this way. This raises a number of possibilities. In a proportion of cases the metastatic disease may represent a dedifferentiated clone of the primary SCLC tumour not expressing somatostatin receptors. Local factors may also be playing a role in individual patients either by down-regulating somatostatin receptor expression or, if high local levels of endogenous somatostatin are being produced, by blocking the receptor site, thereby inhibiting visualisation of the disease. Finally, in image-negative metastatic disease it is possible that the SCLC cells themselves are not expressing specific somatostatin binding sites. Rather it may be that the primary tumour is being visualised because of uptake of the radiolabel by the local inflammatory response, as activated immune cells are known to express somatostatin receptors (Nakamura *et al.*, 1987; Sreedharan *et al.*, 1989). If this were the case then those patients in whom the metastases are seen may represent the true proportion of patients with metastatic SCLC tumours that express high-affinity binding sites for somatostatin – 50% of patients with extensive disease evaluated in this study. This would be in keeping with the known *in vitro* data.

Novel approaches to therapy in SCLC are currently being assessed and include inhibiting the action of autocrine growth factors such as GRP with GRP antagonists and GRP receptor antibodies (Cuttitta *et al.*, 1987; Macaulay & Carney, 1991; Carney, 1992). Somatostatin analogues are currently being evaluated as possible therapeutic agents in SCLC. *In vitro* studies have demonstrated that somatostatin analogues may inhibit the clonal growth of SCLC cell lines. Somatostatin analogues also inhibit vasoactive intestinal polypeptide-induced cAMP accumulation in SCLC cells (Taylor *et al.*, 1988*b*, 1991). The growth of SCLC xenografts has been inhibited in athymic nude mice (Taylor *et al.*, 1988*b*; Bogden *et al.*, 1990; Macaulay *et al.*, 1991). These results suggest that SCLC somatostatin receptors are functional and that the growth-inhibitory effect is direct through inhibition of specific growth pathways within the tumour cells. Somatostatin analogues may also have indirect growth-inhibitory effects through the inhibition of growth factors released from other tissues, such as IGF-1 and epidermal growth factor (Ghirlanda *et al.*, 1983; Macaulay *et al.*, 1991; Damstrup *et al.*, 1992).

Imaging of SCLC tumours with [$^{111}$In]pentetreotide may have a role to play in identifying those patients most likely to respond to somatostatin analogue therapy. Of great significance in this regard is the efficacy of this agent in detecting residual SCLC disease, suggesting that treated SCLC tumours continue to express somatostatin receptors. This lays the groundwork for evaluation of somatostatin analogues as therapeutic agents in the treatment of chemotherapeutically debulked SCLC disease in the future.

The possibility of using radiolabelled antibodies to treat a wide range of tumours has yielded interesting data (Larson, 1987; Goldenberg, 1991). However, one of the principal problems encountered includes the generation of host antibody response to the administered agent rendering the radiolabel useless (Goldenberg, 1991). The possibility of employing a radiolabelled chelated somatostatin analogue as a radiotherapeutic agent in treating somatostatin receptor-positive SCLC tumours is an exciting prospect for the future. As somatostatin analogues are based on sequences of host circulating hormone they rarely induce immunisation (Krenning *et al.*, 1993). The accumulation of [$^{111}$In]pentetreotide in gastrointestinal APUDomas is between 0.0123% and 0.2% of the administered dose per gram of tumour tissue. The rapid clearance of the radiolabel from the blood, the relatively low accumulation in the liver (1.9% and 2.2% of the administered dose at 4 and 24 h respectively) with resultant relatively low excretion into the gastrointestinal tract, and, finally, the predominant renal clearance are advantageous in this regard. However, the amount of renal accumulation and the relatively long renal effective half-life will limit the maximally applicable radiation dose. Studies are required to investigate how to lower the renal radioactivity (Krenning *et al.*, 1993).

In conclusion, (a) the imaging of all disease sites at a single sitting in a significant proportion of patients with extensive disease, thereby making further investigations unnecessary, (b) the localisation of otherwise unexpected metastatic deposits and (c) the detection of residual disease not found by other means suggest that [$^{111}$In]pentetreotide may be a useful adjunct in the diagnostic evaluation of patients with SCLC.

We would like to thank Ms C. O'Donnell, Department of Radiology and Dr R. O'Regan, Department of Oncology, Mater Misericordiae Hospital. We are also indebted to Dr N. O'Hare, Ms Angela Montgomery, Ms Pauline Malone and Ms Helen Ryder of the Nuclear Medicine Department, St. James's Hospital, without whose assistance this work would not have been possible. This study was supported by the Irish Cancer Society.

### References

BEPLER, G., ROTSCH, M., JAQUES, G., HAEDER, M., HEYMANNS, J., HARTOGH, G., KIEFER, P. & HAVEMANN, K. (1988). Peptides and growth factors in small cell lung cancer: production, binding sites, and growth effects. *Cancer Res. Clin. Oncol.*, **114**, 235–244.

BOGDEN, A.E., TAYLOR, J.E., MOREAU, J.-P., COY, D.H. & LEPAGE, D.J. (1990). Response of human lung xenografts to treatment with a somatostatin analogue (somatuline). *Cancer Res.*, **50**, 4360–4365.

CARNEY, D.N. (1992). Biology of small-cell lung cancer. *Lancet*, **339**, 843–849.

CUTTITTA, F., CARNEY, D.N., MULSHINE, J., MOODY, T.W., FEDORKO, J., FISCHLER, A. & MINNA, J.D. (1985). Bombesin-like peptides can function as autocrine growth factors in human small-cell lung cancer. *Nature*, **316**, 823–826.

DAMSTRUP, L., RYGAARD, K., SPANG-THOMSEN, M. & SKOVGAARD POULSEN, H. (1992). Expression of epidermal growth factor receptor in human small cell lung cancer. *Cancer Res.*, **52**, 3089–3093.

GHIRLANDA, G., UCCIOLI, L., PERRI, F., ALTOMONTE, L., BERTOLI, A., MANNA, R., FRATI, L. & GRECO, A.V. (1983). Epidermal growth factor, somatostatin, and psoriasis. *Lancet*, **1A**, 65.

GOLDENBERG, D.M. (1991). Challenges to the therapy of cancer with monoclonal antibodies. *J. Natl Cancer Inst.*, **83**, 78–79.

HANSEN, H.H. (1992). Management of small-cell cancer of the lung. *Lancet*, **339**, 846–849.

HARDY, J., SMITH, I., CHERRYMAN, G., VINCENT, M., JUDSON, I., PERREN, T. & WILLIAMS, M. (1990). The value of computed tomographic (CT) scan surveillance in the detection and management of brain metastases in patients with small lung cancer. *Br. J. Cancer*, **62**, 684–686.

KRENNING, E.P., BAKKER, W.H., BREEMAN, W.A.P., KOPER, J.W., KOOIJ, P.P.M., AUSEMA, L., LAMERIS, J.S., REUBI, J.-C. & LAMBERTS, S.W.J. (1989). Localisation of endocrine-related tumours with radioiodinated analogue of somatostatin. *Lancet*, **i**, 242–244.

KRENNING, E.P., BAKKER, W.H., KOOIJ, P.P., BREEMAN, W.A., OEI, H.Y., DE-JONG, M., REUBI, J.-C., VISSER, T.J., BRUNS, C., KWEKKEBOOM, D.J., REIJS, A.E.M., VAN HAGEN, P.M., KOPER, J.W. & LAMBERTS, S.W.J. (1992*a*). Somatostatin receptor scintigraphy with Indium-111-DTPA-D-Phe-1-octreotide in man: metabolism, dosimetry and comparison with iodine-123-Tyr-3-octreotide. *J. Nucl. Med.*, **33**, 652-658.

KRENNING, E.P., KWEKKEBOOM, D.J., REUBI, J.-C., VAN HAGEN, P.M., VAN EIJCK, C.H.J., OEI, H.Y. & LAMBERTS, S.W.J. (1992*b*). $^{111}$In-octreotide scintigraphy in oncology. *Metabolism*, **41** (Suppl. 2), 83–86.

KRENNING, E.P., KWEKKEBOOM, J.D., BAKKER, W.H., BREEMAN, W.A.P., KOOIJ, P.P.M., OEI, H.Y., VAN HAGEN, M., POSTEMA, P.T.E., DE JONG, M., REUBI, J.-C., VISSER, T.J., REIJS, A.E.M., HOFLAND, L.J., KOPER, J.W., LAMBERTS, S.W.J. (1993). Somatostatin receptor scintigraphy with [$^{111}$In-DTPA-D-Phe$^{1}$]-and[$^{123}$I-Tyr$^{3}$]-octreotide: the Rotterdam experience with more than 1000 patients. *Eur. J. Nucl. Med.*, **20**, 716–731.

KWEKKEBOOM, D.J., KRENNING, E.P., BAKKER, W.H., YOU OEI, H., SPLINTER, T.A.W., SIANG KHO, G. & LAMBERTS, S.J.W. (1991). Radioiodinated somatostatin analog scintigraphy in small-cell lung cancer. *J. Nucl. Med.*, **32**, 1845–1848.

LAMBERTS, S.W.J., BAKKER, W.H., REUBI, J.C. & KRENNING, E.P. (1990*a*). Somatostatin-receptor imaging in the localisation of endocrine tumors. *N. Engl. J. Med.*, **323**, 1246–1249.

LAMBERTS, S.W.J., HOFLAND, L.J., VAN KOETSVELD, P.M., REUBI, J.-C., BRUINING, H.A., BAKKER, W.H. & KRENNING, E.P. (1990*b*). Parallel *in vivo* and *in vitro* detection of functional somatostatin receptors in human endocrine pancreatic tumors: Consequences with regard to diagnosis, localisation and therapy. *J. Clin. Endocrinol. Metab.*, **71**, 566–574.

LARSON, S.M. (1987). Lymphoma, melanoma, colon cancer: diagnosis and treatment with radiolabelled monoclonal antibodies. *Radiology*, **165**, 297–304.

MACAULAY, V.M. & CARNEY, D.N. (1991). Neuropeptide growth factors. *Cancer Invest.*, **9**, 659–673.

MACAULAY, V.M., EVERARD, M.J., TEALE, J.D., TROTT, P.A., VAN WYK, J.J., SMITH, I.E. & MILLAR, J.L. (1990). Autocrine function for insulin-like growth factor 1 in human small cell lung cancer cell lines and fresh tumor cells. *Cancer Res.*, **50**, 2511–2517.

MACAULAY, V.M., SMITH, I.E., EVERARD, M.J., TEALE, J.D., REUBI, J.-C. & MILLAR, J.L. (1991). Experimental and clinical studies with somatostatin analogue octreotide in small cell lung cancer. *Br. J. Cancer*, **64**, 451–456.

MALEC, P., ZEMAN, K., MARKIEWICZ, K., TCHORZEWSKI, H., NOWAK, Z. & BAJ, Z. (1989). Short-term somatostatin infusion affects T lymphocyte responsiveness in humans. *Immunopharmacology*, **17**, 45–49.

MESCARDO, R.N. & SHERLINE, P. (1982). Somatostatin inhibits rapid centrosomal separation and cell proliferation induced by epidermal growth factor. *Endocrinology*, **82**, 1394–1396.

MINNA, J.D., PASS, H., GLATSTEIN, E. & IHDE, D.C. (1989). Cancer of the lung. In *Cancer: Principles and Practice of Oncology*, 3rd edn, DeVita, V.T., Hellman, S. & Rosenberg, S.A. (eds) pp. 631–636, 666–687. Philadelphia: Lippincott.

MOODY, T.W., CARNEY, D.N., CUTTITTA, F., QUATTROCCHI, K., MINNA, J.D. (1985). High affinity receptors for bombesin/GRP-like peptides on human small cell lung cancer. *Life Sci.*, **37**, 105–113.

NAKAMURA, H., KOIKE, T., HIRUMA, K., SATO, T., TOMIOKA, H. & YOSHIDA, S. (1987). Identification of lymphoid cell lines bearing receptors for somatostatin. *Immunology*, **62**, 655–658.

REICHLIN, S. (1983*a*). Somatostatin. Part I. *N. Engl. J. Med.*, **309**, 1495–1501.

REICHLIN, S. (1983*b*). Somatostatin. Part II. *N. Engl. J. Med.*, **309**, 1556–1563.

REUBI, J.-C., KVOLS, L., KRENNING, E. & LAMBERTS, S.W.J. (1990*a*). Distribution of somatostatin receptors in normal and tumor tissue. *Metabolism*, **39** (9 Suppl. 2), 78–81.

REUBI, J.-C., WASER, B., SHEPPARD, M. & MACAULAY, V. (1990*b*). Somatostatin receptors are present in small-cell but not in non-small-cell primary lung carincomas: relationship to EGF-receptors. *Int. J. Cancer*, **45**, 269–274.

SAGMAN, U., MULLEN, B., KOVACS, K., KERBEL, R., GINSBERG, R. & REUBI, J.-C. (1990). Identification of somatostatin receptors in human small cell lung carcinoma. *Cancer*, **66**, 2129–2133.

SHEPPARD, M.N. (1991). New perspectives in lung cancer. 1. Neuroendocrine differentiation in lung tumours. *Thorax*, **46**, 843–850.

SREEDHARAN, S.P., KODAMA, K.T., PETERSON, K.E. & GOETZL, E.J. (1989). Distinct subsets of somatostatin receptors on cultured human lymphocytes. *J. Biol. Chem.*, **264**, 949–952.

TAYLOR, J.E., COY, D.H. & MOREAU, J.-P. (1988*a*). High affinity binding of [$^{125}$I-Tyr$^{11}$]somatostatin-14 to human small-cell carcinoma (NCI-H69). *Life Sci.*, **43**, 421–427.

TAYLOR, J.E., BOGDEN, A.E., MOREAU, J.-P. & COY, D.H. (1988*b*). *In vitro* and *in vivo* inhibition of small cell lung carcinoma (NCI-H69) growth by a somatostatin analogue. *Biochem. Biophys. Res. Commun.*, **153**, 81–86.

TAYLOR, J.E., MOREAU, J.P., BAPTISTE, L. & MOODY, T.W. (1991). Octapeptide analogues of somatostatin inhibit the clonal growth and vasoactive intestinal peptide-stimulated cyclic AMP formation in human small cell lung cancer. *Peptides*, **12**, 839–843.

VAN HAGEN, P.M., KRENNING, E.P., REUBI, J.C., MULDER, A.H., BAKKER, W.H., OEI, H.Y., LOWENBERG, B. & LAMBERTS, S.W.J. (1993). Somatostatin analogue scintigraphy of malignant lymphomas. *Br. J. Haematol.*, **83**, 75–79.

VEHMEYER, K., SCHUFF-WERNER, P. & NAGEL, G.A. (1986). Suppressed colony formation of peripheral blood lymphocytes in a patient with somatostatinoma. *Clin. Immunol. Immunopathol.*, **41**, 290–294.